# Waist‐To‐Hip Ratio Is More Predictive of Patients‐Reported Outcomes After Total Joint Arthroplasty Than Body Mass Index: A Prospective Cohort Study

**DOI:** 10.1111/os.70201

**Published:** 2025-10-25

**Authors:** Long Zhao, Yinghao Wang, Duan Wang, Zongke Zhou

**Affiliations:** ^1^ Department of Orthopedics and Orthopedic Research Institute West China Hospital, Sichuan University Chengdu People's Republic of China

**Keywords:** body mass index (BMI), total knee arthroplasty (TKA), waist‐to‐hip ratio (WHR)

## Abstract

**Objectives:**

Obesity has an important impact on the future of total joint arthroplasty (TKA). We aimed to determine whether waist‐to‐hip ratio (WHR) is a useful measurement in predicting postoperative outcomes associated with obesity in patients undergoing primary TKA and compared the predictive value of WHR to that of body mass index (BMI).

**Methods:**

Prospective data from patients undergoing unilateral primary TKA from February to May 2024 were analyzed, including BMI and WHR. Outcomes included complications, hospitalization details, and 12‐month patient‐reported function (University of California, Los Angeles [UCLA] activity scale, the Hospital for Special Surgery [HSS] score). Multivariable regression models were used to identify significant obesity‐related predictors of outcomes.

**Results:**

A total of 195 patients were included, with the mean BMI of 28.2 ± 5.2 kg/m^2^ (range: 17.6–40.8) and the mean WHR of 1.03 ± 0.08 (range: 0.83–1.27). WHR was a significant predictor of wound complication (OR: 1.087, *p* = 0.016). Both WHR (OR: 1.153, *p* = 0.004) and BMI (OR: 1.058, *p* = 0.021) independently predicted systemic complications, with WHR explaining greater variance ( *R* ^2^ = 0.241 vs. 0.107 for BMI). For functional outcomes, higher WHR was associated with poorer UCLA activity scores (RR: 0.877, *p* = 0.012) and HSS function scores (RR: 0.921, *p* < 0.001), whereas BMI only showed significance for HSS function scores (RR: 0.960, *p* = 0.002). WHR again explained more variance in HSS function scores ( *R* ^2^ = 0.233 vs. 0.124). In contrast, neither WHR nor BMI correlated with surgical records, hospitalization days, or HSS pain scores (all *p* > 0.05).

**Conclusions:**

The WHR demonstrates superior predictive value over BMI for perioperative complications and 12‐month patient‐reported functional outcomes following primary TKA. Preoperative WHR assessment may help surgeons improve risk stratification and better educate obese patients regarding postoperative expectations prior to elective TKA.

## Graphical abstract

Our findings highlight that the incorporation of preoperative WHR evaluation into clinical practice may assist surgeons in preoperative risk warning, patient education and surgical timing decisions for obese candidates considering elective total knee arthroplasty.

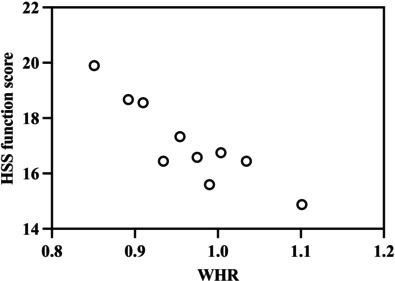

## 1 Introduction

The prevalence of obesity continues to increase, with an estimated one‐eighth of the world's population now affected [1]. Obesity and its biomechanical and physiological effects have been shown to increase the risk of developing knee osteoarthritis, which consequently leads to a rising demand for total knee arthroplasty (TKA) among obese individuals [2, 3, 4]. The risks associated with performing TKA in obese patients are well‐recognized, including increased operative time, periprosthetic infections, and decreased physical conditioning [5, 6, 7]. Nevertheless, obese patients often experience substantial pain relief and improved function following TKA [8]. It is essential to clearly define potential risk factors and explain these risks to patients before performing elective TKA. Conceivably, a superior preoperative identifier of obesity‐related risk factors would improve patient counseling regarding arthroplasty risks and expectations.

Body mass index (BMI, which classifies overweight as ≥ 24 kg/m^2^ and obesity as ≥ 28 kg/m^2^) [9], remains a prevailing proxy for obesity based on the relationship between height and weight. However, it is unable to distinguish fat mass from muscle mass or central adiposity from peripheral adiposity. These imperfections in what has long been the standard of body habitus characterization can introduce bias into sensitivity and specificity estimates when analyzing its association with adverse postoperative outcomes, leading to contradictory conclusions [10, 11, 12]. In contrast, waist‐to‐hip ratio (WHR), calculated as the ratio of waist circumference to hip circumference, is a patient‐specific measure that characterizes body fat mass and its distribution. A higher WHR (≥ 0.90 in men; ≥ 0.85 in women) indicates central obesity with excess abdominal fat and reduced lower‐extremity muscle mass [9]. Evidence suggests WHR is a more robust indicator of metabolic dysfunction and mechanical overload than BMI [13], and therefore may better predict patient‐reported outcomes following lower‐extremity arthroplasty, particularly TKA.

Currently, limited data are available for predicting postoperative outcomes after TKA using WHR. To address this gap, we embarked on this prospective cohort study (i) to evaluate WHR's utility in predicting obesity‐associated clinical and functional outcomes following TKA, and (ii) to compare its predictive performance with that of BMI. Given that WHR is a more informative measure than BMI, we hypothesized that WHR would outperform BMI in predicting clinical risks and patient‐reported functional outcomes after TKA.

## 2 Patients and Methods

### 2.1 Study Design and Setting

The study was carried out according to the ethical principles of the Helsinki Declarations. Approval was granted by the Clinical Trials and Biomedical Ethics Committee of our institution (No. 2024‐92). All participants provided written informed consent. The study was registered in the Chinese Clinical Trial Registry (registration number: ChiCTR2400087646).

### 2.2 Patients

**FIGURE 1:**
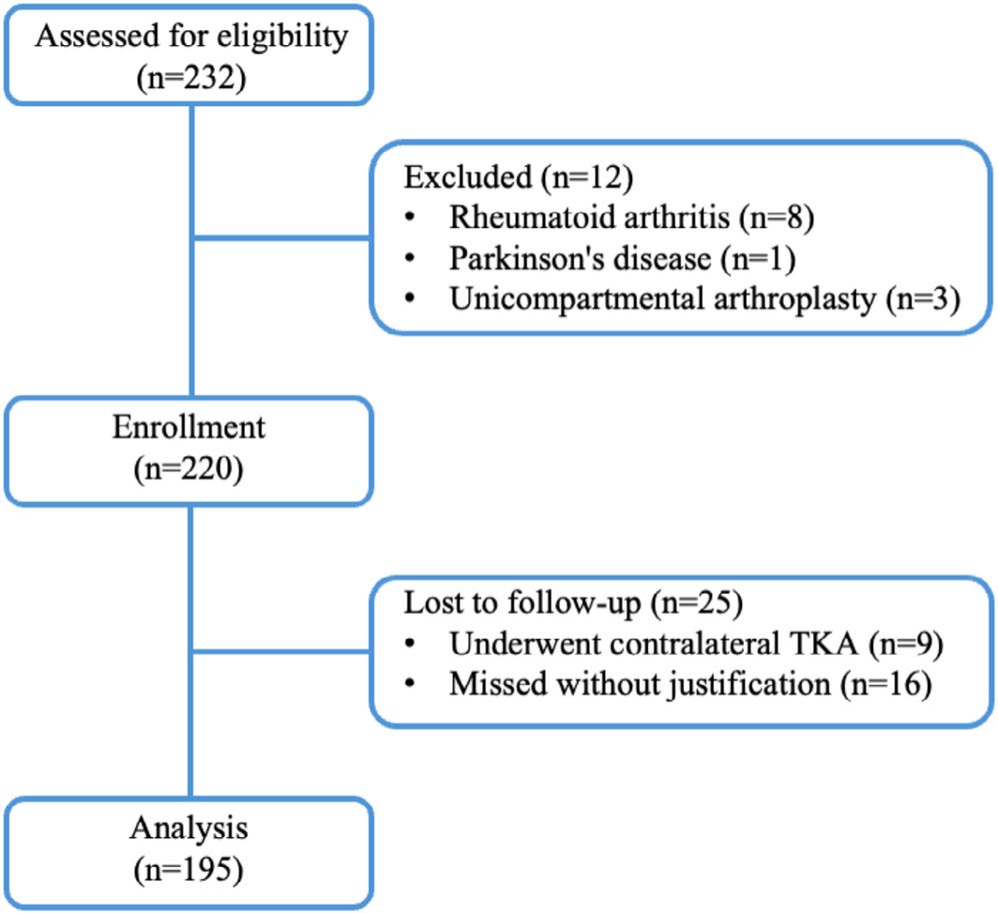
Flowchart of patient screening and follow‐up.

An a priori sample size calculation was performed for a linear multiple regression model (*R* ^2^ increase), with *α* = 0.05, power = 0.80, and a small‐to‐medium effect size (*f* ^2^ = 0.10). Accounting for one tested predictor and three covariates, the minimum sample required was 176. To accommodate a 25% dropout rate, we prospectively enrolled a cohort of 220 patients aged 40–80 years who underwent primary unilateral TKA for osteoarthritis at our institution from February to May 2024. The study enrolled a consecutive cohort of patients based on strict clinical criteria: inclusion was limited to those aged 40–80 undergoing primary TKA for advanced osteoarthritis (Kellgren–Lawrence grade 3–4), while exclusion criteria comprised secondary causes of arthritis (e.g., rheumatoid, post‐traumatic), significant deformities (> 30°), neurological deficits, and prior arthroplasty (Figure 1).

### 2.3 Obesity Evaluation

Preoperative height and weight were measured to calculate each patient's BMI. Additionally, waist and hip circumferences were measured preoperatively to determine the WHR. These measurements were taken using a flexible, inextensible measuring tape with an accuracy of 1 mm. The waist circumference was measured on a horizontal plane at a point equidistant from the lowest floating rib and the upper border of the iliac crest. During the measurement, the patient stood upright, and the measurement was taken after exhaling but before inhaling. The hip circumference was measured on a horizontal plane at the maximum protuberance of the buttocks, which coincides in the front with the ischiopubic symphysis. Each measurement was repeated three times and the mean value was recorded. Measurement reliability analysis demonstrated excellent intra‐observer consistency for WHR, with intraclass correlation coefficients of 0.81 (95% CI: 0.76–0.86) for waist circumferences and 0.83 (95% CI: 0.75–0.89) for hip circumferences.

### 2.4 Perioperative Management

All patients underwent general anesthesia. After preoxygenation, anesthesia was induced with sufentanil (0.3 μg/kg), cisatracurium (0.2 mg/kg), propofol (2 mg/kg), and midazolam (2 mg/kg), and maintained with sufentanil (0.1 μg/kg), atracurium (0.5 mg/kg), and sevoflurane (1%–3%). All procedures were performed by three fellowship‐trained arthroplasty specialists through a medial parapatellar approach. The surgical technique combined measured resection for femoral component positioning with gap balancing to optimize soft‐tissue tension. Femoral preparation used an intramedullary guide, while tibial resection employed an extramedullary alignment system. All cemented femoral and tibial components were implanted without patellar resurfacing. All patients were prescribed a tailored exercise program commencing on postoperative Day 1, focusing on enhancing muscular strength, increasing joint range of motion, and improving walking ability.

### 2.5 Data Collection

Assessments were performed at hospitalization, 1, 3, 6, and 12 months postoperatively, with evaluators blinded to participants' WHR and BMI data. The analyzed dataset contained comprehensive patient information: baseline clinicodemographic characteristics (including age, sex, American Standard Association [ASA] score, BMI and WHR), detailed perioperative documentation (including surgery duration, bleeding, hospitalization days, and systemic complications that required medical intervention), and outpatient follow‐up records that systematically tracked wound complications and patient‐reported outcomes. Patient‐reported outcomes were assessed at 12‐month follow‐up using two standardized questionnaires: the University of California, Los Angeles (UCLA) activity scale and the Hospital for Special Surgery (HSS) scales. The UCLA scale measures subjective activity levels across 10 tiers, from wholly inactive (level 1) to regular impact sports (level 10), and is the preferred functional assessment tool for arthroplasty patients [14]. In contrast, the HSS scales provide an objective evaluation of functional outcomes, mainly focusing on joint pain, range of motion, and activities of daily living. It demonstrates excellent internal consistency (Cronbach's *α* = 0.89) and test–retest reliability (ICC = 0.91) [15].

### 2.6 Statistical Analysis

Routine descriptive statistics were computed, including continuous variables summarized as means and standard deviations, and categorical variables expressed as counts and percentages. Multivariable analyses employed generalized regression models to identify obesity‐related predictors (BMI and WHR) of clinical and functional outcomes, adjusting for prespecified covariates (sex, age, comorbidities). For outcomes significantly associated with obesity indicators, we generated risk decile plots to explore potential threshold effects. Dichotomous outcomes were analyzed via logistic regression (reporting adjusted odds ratio [OR]), while count data used generalized linear models (reporting risk ratio [RR] per 1‐SD increase in BMI or WHR). All estimates included 95% confidence interval (CI). Model fit was assessed using *R* ^2^, and linear correlations via Pearson coefficients. Analyses were performed in SPSS 29.0 (IBM), with significance at *p* < 0.05 (two‐tailed).

## 3 Result

### 3.1 General Clinical‐Demographic Results

A total of 25 patients were lost to follow‐up (9 received contralateral TKA within the follow‐up period, and 16 missed visits without justification) and were excluded from analysis; given that their baseline characteristics were comparable to the retained cohort (*p* > 0.05 for all demographics, indicating random missingness), this exclusion was unlikely to introduce bias. The final analytic cohort included 195 patients. This cohort comprised 57 males (29.2%) and 138 females (70.8%), with a mean age of 67.2 ± 7.9 years. Mean BMI was 28.2 ± 5.2 kg/m^2^ (range: 17.6–40.8), and mean WHR was 1.03 ± 0.08 (range: 0.83 to 1.27). Based on BMI criteria (≥ 28.0 kg/m^2^), 43.1% (84/195) were classified as obesity, while 71.8% (140/195) met the criteria for central obesity (WHR ≥ 0.90 for males and ≥ 0.85 for females). Moreover, 73.9% (82/111) of BMI‐defined nonobese patients had central obesity, and 14.3% (12/84) of BMI‐defined obese patients exhibited noncentral obesity (Table 1).

**TABLE 1:** Patient baseline clinicodemographic characteristics.

Characteristics	Outcomes
Age (years) (SD)^a^	67.2 (7.9)
Sex (male:female)^b^	57:138
Height (cm) (SD)^a^	156.4 (8.8)
Weight (kg) (SD)^a^	72.3 (12.0)
BMI (kg/m^2^) (SD)^a^	28.2 (5.2)
Hipline (cm) (SD)^a^	97.9 (5.4)
Waistline (cm) (SD)^a^	92.9 (11.0)
WHR^a^	1.03 (0.08)
ASA score^b^	
I	17
II	143
III	35

^a^ The values are presented as mean (standard deviation, SD).

^b^ The values are presented as the number (percentage) of patients.

### 3.2 Prediction in Post‐TKA Medical Complications

In multivariable analyses, both WHR (OR: 1.153; 95% CI: 1.033–1.821; *p* = 0.004) and BMI (OR: 1.058; 95% CI: 1.011–1.444; *p* = 0.021) were significant predictors of systemic complications, including acute kidney injury (two patients), acute liver dysfunction (*n* = 3), cardiac dysfunction (eight patients), respiratory or urinary tract infections (five patients), non‐infectious fever (11 patients), and symptomatic pulmonary embolism (one patient). Notably, WHR showed greater explanatory power for the risk of these events (*R* ^2^ = 0.241) than BMI (*R* ^2^ = 0.107), with each one–standard‐deviation increase in WHR and BMI being associated with a 15.3% and 5.8% higher risk of systemic complications, respectively. For wound complications (eight cases of oozing and three of delayed healing), only WHR was a significant predictor (OR: 1.087; 95% CI: 1.025–1.421; *p* = 0.016; *R* ^2^ = 0.335), corresponding to an 8.7% higher risk per SD increase (Table 2). Neither WHR nor BMI predicted surgical bleeding, duration, or hospitalization (Table 3).

**TABLE 2:** Prediction of dichotomous clinical outcomes after adjustment for significant baseline demographic covariates.

Outcome and predictor	Outcomes^a^	Odds ratio^b^	*R* ^2^	*p*
Systemic complications				
BMI	30:165	1.058 (1.011–1.444)	0.107	0.021^c^
WHR		1.153 (1.033–1.821)	0.241	0.004^c^
Wound complications				
BMI	11:184	0.926 (0.627–1.806)	0.094	0.158
WHR		1.087 (1.025–1.421)	0.335	0.016

^a^ Data are presented as the ratio of “yes” to “no” outcomes.

^b^ Values represent odds ratios per one standard deviation increase in the covariate, accompanied by 95% confidence intervals in parentheses.

^c^ Indicates statistical significance at the *p* < 0.05 level.

**TABLE 3:** Prediction of count data for clinical and patient‐reported functional outcomes after adjustment for significant baseline demographic covariates.

Outcome and predictor	Outcomes^a^	Risk ratio^b^	*R* ^2^	*p*
Surgical time (minutes)				
BMI	82.22 (28.10)	0.966 (0.933–1.034)	—	0.530
WHR		1.067 (0.873–1.184)	—	0.325
Surgical bleeding (mL)				
BMI	80.57 (45.67)	1.101 (0.655–1.647)	—	0.875
WHR		1.037 (0.902–1.335)	—	0.429
Hospitalization (days)				
BMI	5.86 (1.31)	1.142 (0.814–1.320)	—	0.354
WHR		1.233 (0.900–1.431)	—	0.400
UCLA activity score				
BMI	4.04 (1.25)	0.922 (0.821–1.013)	—	0.228
WHR		0.877 (0.811–0.962)	0.217	0.012^c^
HSS function score				
BMI	18.44 (2.92)	0.960 (0.913–0.995)	0.124	0.002^c^
WHR		0.921 (0.883–0.971)	0.233	< 0.001^c^
HSS pain score				
BMI	29.21 (3.472)	0.981 (0.888–1.214)	—	0.173
WHR		0.900 (0.830–1.012)	—	0.094

^a^ The values are given as mean (standard deviation, SD).

^b^ The values are given as the risk ratio per one standard deviation increase in the covariate, with the 95% CI in parentheses.

^c^ Predictor was significant at *p* < 0.05.

### 3.3 Prediction in Post‐TKA Functional Outcomes

For patient‐reported functional outcomes, WHR demonstrated significant predictive value for the UCLA activity score (RR: 0.877; 95% CI: 0.811–0.962; *p* = 0.012; *R* ^2^ = 0.217), with a 12.3% reduction in UCLA activity score per standard deviation increase in WHR. In contrast, BMI failed to show a significant association with UCLA activity scores. Regarding the HSS function score, both obesity indicators emerged as significant predictors; WHR (RR: 0.921; 95% CI: 0.883–0.971; *p* < 0.001) demonstrated greater explanatory power (*R* ^2^ = 0.233) compared to BMI (RR: 0.960; 95% CI: 0.913–0.995; *p* = 0.002; *R* ^2^ = 0.124), with each one standard deviation increase in WHR and BMI associated with an8.9% and 4.0% decrease in HSS function score, respectively. Additionally, WHR showed a trend toward significance in predicting HSS pain scores (RR: 0.900; 95% CI: 0.830–1.012; *p* = 0.094), while BMI demonstrated no such association (Table 3).

**FIGURE 2:**
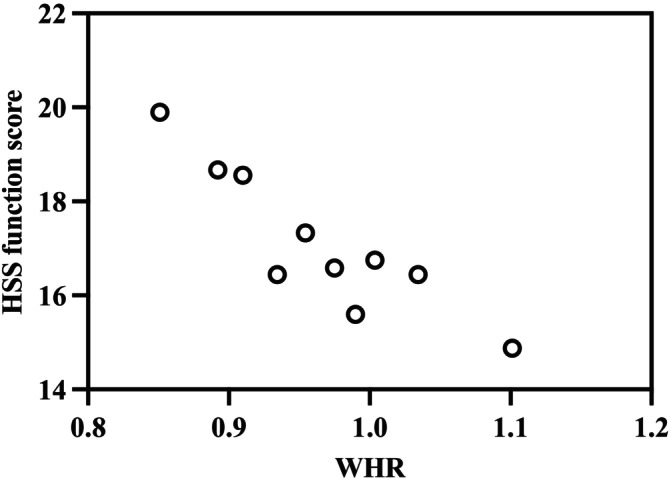
Significant linear relationship between waist‐to‐hip ratio (WHR) and the Hospital for Special Surgery (HSS) score following total knee arthroplasty (plotted for each WHR decile). Pearson correlation coefficient = −0.518 (*p* < 0.001). *N* = 195.

**FIGURE 3:**
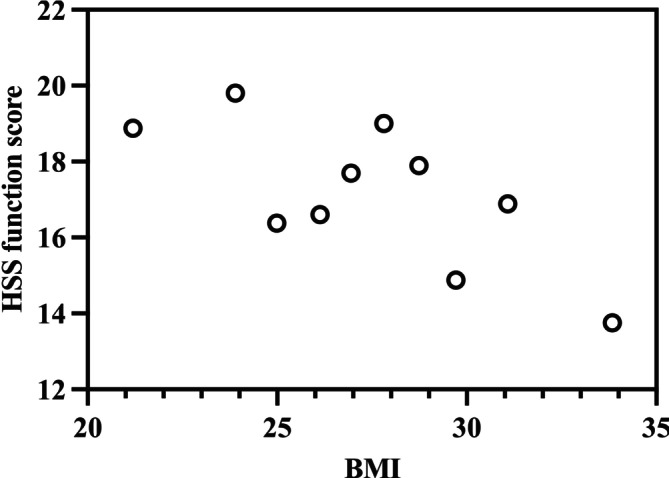
Significant linear relationship between body mass index (BMI) and the Hospital for Special Surgery (HSS) score following total knee arthroplasty (plotted for each BMI decile). Pearson correlation coefficient = −0.431 (*p* < 0.001). *N* = 195.

**FIGURE 4:**
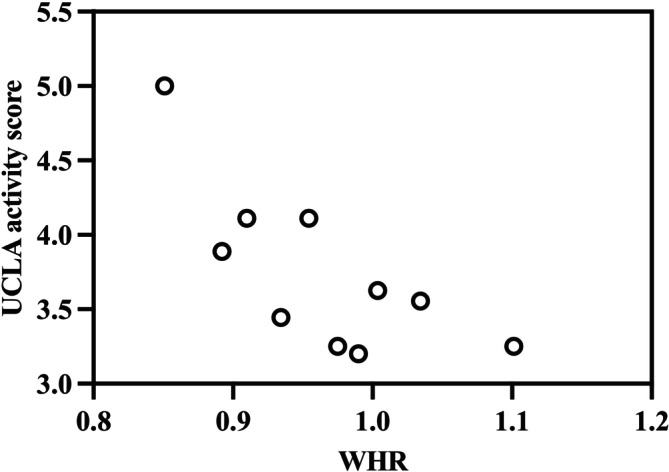
Significant linear relationship between waist‐to‐hip ratio (WHR) and the University of California, Los Angeles (UCLA) activity score following total knee arthroplasty (plotted for each WHR decile). Pearson correlation coefficient = −0.326 (*p* < 0.001). *N* = 195.

Additional analyses were conducted to examine obesity indicator predictors of specific clinical outcomes, focusing on identifying potential cut‐off points for BMI and WHR. For systemic complications, significantly higher odds were observed at WHR > 1.07 (OR: 9.128; 95% CI: 2.864–28.710; *p* = 0.042) and BMI > 35.2 (OR: 5.842; 95% CI: 1.663–26.452; *p* = 0.017). For wound complications, significantly higher odds were observed at WHR > 1.15 (OR: 12.72; 95% CI: 1.873–40.284; *p* = 0.035). The HSS functional score exhibited a strong negative correlation with WHR (*r* = −0.518; *p* < 0.001) (Figure 2), with WHR > 1.12 predicting a significantly increased risk ratio (RR: 0.234; 95% CI: 0.024–0.862; *p* < 0.001). Similarly, the HSS functional score showed a significant negative linear relationship with BMI (*r* = −0.431; *p* < 0.001) (Figure 3), although no specific BMI threshold was identified for significantly increased risk. The UCLA activity score also demonstrated a significant negative correlation with WHR (*r* = −0.326; *p* < 0.001) (Figure 4), but no specific WHR cut‐off point was associated with a significantly increased risk ratio.

## 4 Discussion

The detrimental impact of obesity on the outcomes of TKA remains a significant and growing concern for surgeons. Identifying accurate measures of obesity is a crucial prerequisite for setting appropriate expectations regarding arthroplasty risks and benefits in obese patients. Importantly, previous studies have cautioned against relying solely on BMI as an indicator of obesity, due to its limited ability to fully characterize body fat distribution and associated risks [9, 10, 11]. In response to these concerns, we employed the WHR as an obesity indicator to evaluate its predictive capacity for TKA outcomes, while conducting a comparative analysis with the conventional BMI measurement in a prospective cohort study design. The principal findings revealed that WHR demonstrated superior predictive validity over BMI, particularly in forecasting both complications and 12‐month patient‐reported functional outcomes following knee arthroplasty.

### 4.1 WHR Outperforms BMI in Predicting Post‐TKA Medical Complications

Clinically, obesity exhibits extensive heterogeneity in phenotype. Previous studies have reported significant deviations in body composition among individuals within the same obesity categories classified by BMI. These deviations include twofold differences in adiposity and up to 30 kg differences in lean soft tissue [16]. Consistent with these findings, our cohort showed considerable WHR variability within BMI classifications: 74.5% of BMI‐defined nonobese patients had central obesity by WHR, while 12.2% of BMI‐defined obese patients exhibited noncentral obesity. This discrepancy between BMI and WHR in characterizing obesity carries significant clinical implications, particularly in predicting postoperative complications. Evidence suggests that WHR is superior to BMI in predicting complications such as wound infections, reoperations, other medical morbidities, and mortality following abdominal, pelvic and acetabular procedures [17, 18]. These findings are further corroborated by data from patients undergoing TKA. In our cohort, WHR significantly predicted wound complications, whereas BMI did not. Moreover, each standard‐deviation increase in WHR was associated with a 15.3% higher risk of systemic complications—more than 2.5 times the effect size of BMI (5.8%). These results underscore the importance of incorporating WHR assessment, rather than relying solely on BMI, into future risk prediction models for patients undergoing TKA.

### 4.2 WHR Outperforms BMI in Predicting Post‐TKA Functional Outcomes

Another key motivation for this study was to examine how body fat distribution influences functional outcomes after TKA. Our results demonstrate that WHR outperforms BMI in predicting postoperative functional impairment. For each standard deviation increase, WHR was associated with an 8.9% decline in 12‐month postoperative HSS scores—more than double the effect of BMI (4.0% decline). This enhanced predictive power of WHR likely stems from the biomechanical effects of central adiposity. Previous studies suggest that central obesity, marked by excess abdominal fat, alters the body's center of mass, increasing knee joint moments and extensor muscle demand during daily activities [19]. In contrast, peripheral obesity may exert less mechanical stress on the knees. Postoperatively, these biomechanical challenges may be particularly consequential. Patients typically experience 60%–80% loss of preoperative knee extensor strength due to arthrogenic muscle inhibition—a reflex suppression of muscle activation following joint injury [20, 21]. When compounded by the additional joint stress from central obesity, this muscle weakness could further hinder functional recovery. Our findings underscore that WHR—a measure of fat distribution—holds greater clinical relevance than BMI alone in forecasting functional outcomes after TKA.

Beyond biomechanics imbalance, elevated WHR is also linked to inflammatory dysfunction driven by visceral fat accumulation. Growing evidence confirms important biological distinctions between visceral and subcutaneous adipose tissue. Specifically, visceral fat and its associated macrophages demonstrate heightened secretion of pro‐inflammatory cytokines (including interleukin‐6, C‐reactive protein, and tumor necrosis factor α), establishing a chronic low‐grade inflammatory milieu [22, 23]. This persistent inflammatory state has been identified as a potential direct contributor to chronic residual pain and compromised joint function following TKA [24, 25]. Although our current study did not include serum inflammatory cytokine analysis, we postulate that the augmented postoperative inflammatory response may represent a plausible mechanism underlying the poor clinical outcomes associated with elevated WHR. This interpretation is supported by robust evidence demonstrating that central obesity confers greater metabolic and postoperative inflammatory risks than peripheral obesity [26, 27].

## 5 Limitation and Strengths

Several study limitations warrant consideration when interpreting our findings. First, as our study included only single‐center osteoarthritis patients with short follow‐up, the findings may lack generalizability to other TKA populations and require validation in diverse clinical settings with longer follow‐up. Second, while our analysis focused on mild‐to‐moderate obesity cases, future studies should incorporate the full spectrum of obesity severity to enhance clinical applicability. Third, although we adjusted for major confounders (including sex, age, and ASA physical status), residual confounding may persist due to unmeasured variables such as socioeconomic status. Finally, our reliance on single baseline WHR measurements prevents assessment of longitudinal body composition changes—an important area for future research examining temporal variations in adiposity metrics and their relationship with TKA outcomes.

Our study also has several notable strengths. First, to our knowledge, this is the first investigation to comprehensively compare the predictive utility of WHR and BMI specifically within a TKA population, providing novel insights into optimal obesity assessment in this surgical context. Second, we utilized a rigorously compiled prospective registry, ensuring high‐quality and detailed data collection for all outcomes, and confounding variables. Finally, the evaluation of multiple functional outcomes enhances the clinical relevance of our findings, offering a more holistic view of the postoperative recovery experience for patients.

Despite these limitations, our study establishes WHR as a clinically superior obesity indicator compared to BMI for preoperative risk assessment and functional outcome prediction in TKA patients. The incorporation of preoperative WHR evaluation into clinical practice may assist surgeons in preoperative risk warning, patient education and surgical timing decisions for obese candidates considering elective TKA.

## 6 Conclusion

The WHR demonstrates superior predictive value over BMI for perioperative complications and 12‐month patient‐reported functional outcomes following total knee arthroplasty. WHR assessment may help surgeons improve risk stratification, project patient‐reported functional outcomes, and better educate obese patients regarding postoperative expectations prior to elective total knee arthroplasty.

## Author Contributions

**Long Zhao:** conceptualization, writing – original draft. **Yinghao Wang:** methodology, data curation. **Duan Wang:** validation, supervision. **Zongke Zhou:** project administration, supervision.

## Ethics Statement

This prospective study secured approval from the Clinical Trials and Biomedical Ethics Committee of West China Hospital, Sichuan University. The study was registered in the Chinese Clinical Trial Registry (registration number: ChiCTR2400087646).

## Consent

All participants provided written informed consent.

## Conflicts of Interest

The authors declare no conflicts of interest.

## Data Availability

The data that support the findings of this study are available on request from the corresponding author. The data are not publicly available due to privacy or ethical restrictions.
